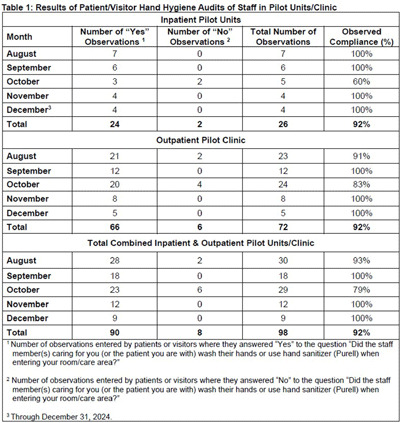# Implementing a Patient-Driven Hand Hygiene Auditing Pilot Program: A Novel Approach to Improving Compliance

**DOI:** 10.1017/ash.2025.335

**Published:** 2025-09-24

**Authors:** Kevin Gibas

**Affiliations:** 1 Rhode Island Hospital

## Abstract

**Background:** Hand hygiene is the most crucial practice for reducing the transmission of infections in healthcare settings. Despite substantial data highlighting its importance, healthcare institutions frequently struggle to maintain high hand hygiene compliance rates among healthcare workers (HCWs). Low HCW hand hygiene compliance can lead to an increase in healthcare-associated infections, longer hospital stays, antimicrobial resistance, and higher healthcare costs. Low compliance can also have regulatory and reimbursement implications for healthcare systems. This highlights the urgent need for innovative interventions focused on improving HCW hand hygiene compliance. **Methods:** We piloted a novel program at Brown University Health (BUH) that enabled patients and visitors to audit HCW hand hygiene using an online interface. This trial was implemented in an iterative fashion in 1 outpatient clinic and 5 inpatient units over 5 months (Figure 1). A poster with program information and a QR code linked to the audit form was placed in each room and/or handed to patients/visitors. Patients/visitors were instructed to scan the QR code with their phones to access the audit form, which included three questions about their location (inpatient/outpatient), if HCWs performed hand hygiene (yes/no), and if they would feel comfortable asking staff to perform hand hygiene (yes/no). The form was available in English, Spanish, and Portuguese. Responses were recorded securely and anonymously in the online platform and monitored by Infection Control. Additionally, HCWs on these units received a survey to provide feedback on this program. **Results:** Patients and visitors recorded 98 hand hygiene audits of staff during the program trial: 72 from the outpatient clinic and 26 from the inpatient units (Table 1). HCW hand hygiene compliance observed by patients/visitors was 92% in both inpatient and outpatient settings, resulting in an overall compliance rate of 92% during the pilot program. Figure 1 shows HCW hand hygiene compliance rates as measured by trained BUH staff in the pilot units and clinic during 2024 in relation to the implementation of this program. We found that after the start of education and implementation of the patient/visitor hand hygiene auditing program in these units, compliance generally increased and remained above pre-intervention levels. **Conclusions:** This pilot program demonstrates the feasibility and potential effectiveness of engaging patients and visitors in hand hygiene interventions. The results of this pilot suggest that this novel approach warrants further investigation and broader implementation as part of larger efforts improve HCW hand hygiene compliance and reduce healthcare-associated infections.

**Figure:**
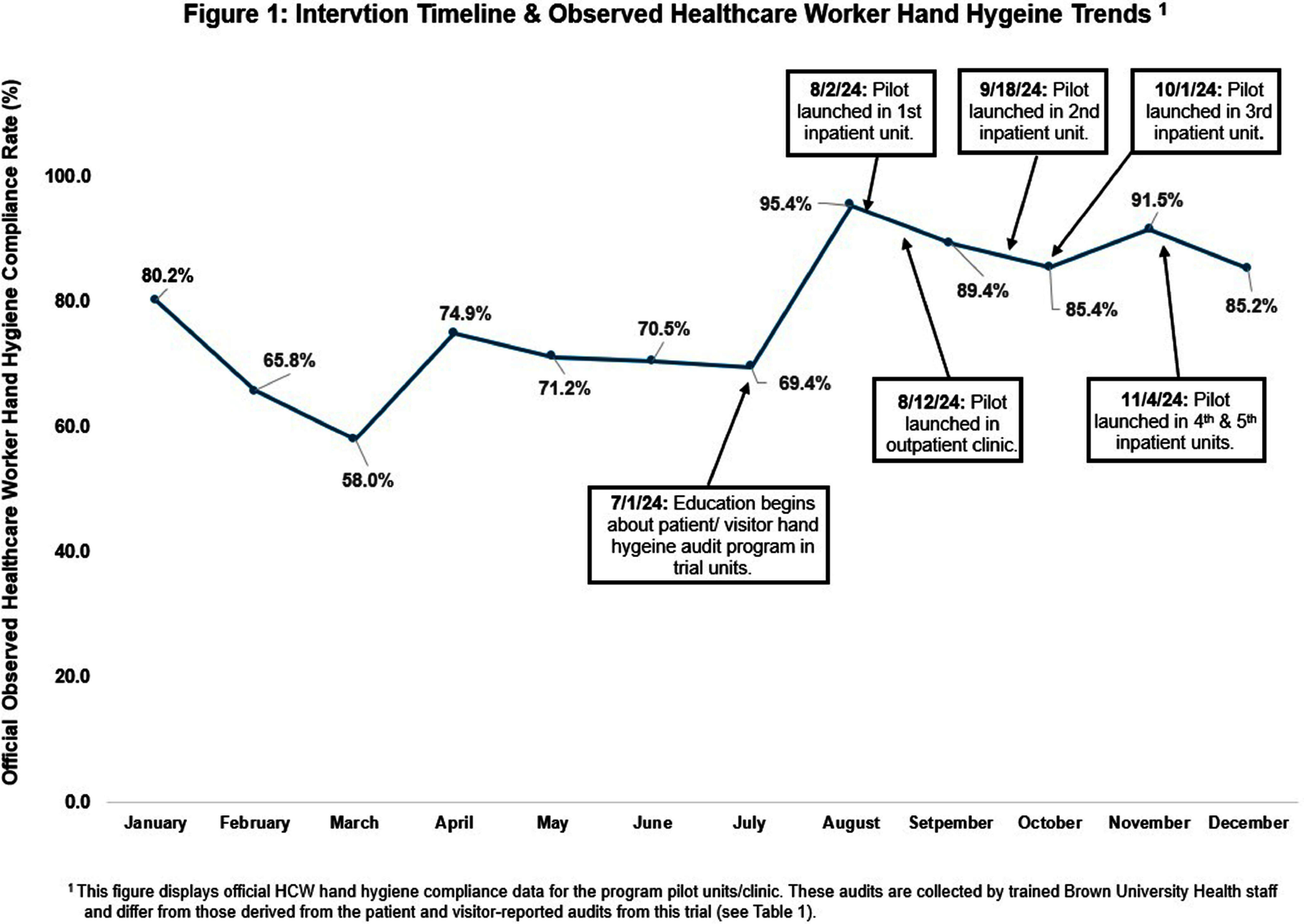

**Figure:**